# Comparative Analysis of Microabrasive Film Finishing Effects across Various Process Variants

**DOI:** 10.3390/ma17143582

**Published:** 2024-07-19

**Authors:** Katarzyna Tandecka, Wojciech Kacalak, Thomas G. Mathia

**Affiliations:** 1 Department of Engineering and Informatics Systems, Faculty of Mechanical Engineering and Energy, Koszalin University of Technology, 75620 Koszalin, Poland; wojciech.kacalak@tu.koszalin.pl; 2 Laboratoire de Tribologie et Dynamique des Systemes (LTDS), Ecole Centrale de Lyon, Centre National de la Recherche Scientifique, 69134 Lyon, France

**Keywords:** surface finishing, abrasive film, finishing, abrasion, superfinishing, superalloy, machining capability, nickel–phosphorus alloy, Ni–P

## Abstract

The paper investigates various methods of microfinishing and arrives at the best technique to produce a very smooth surface. Various setups, with and without oscillation, were developed, together with a microfinishing attachment used on conventional lathes and milling machines. The workpiece material used was an amorphous nickel–phosphorus Ni–P alloy. The surface roughness parameters, such as Sa, Sv, and Sp, were measured with the TalySurf CCI6000 instrument. For the measurement of the surface protrusions, an “analysis of islands” technique was used at various levels of cut-off. The 2BA method—machining below the workpiece axis with oscillation—turned out to be the most effective method applied because it had the highest density of protrusions while having the smallest value of surface roughness. Non-oscillation with the machining zone below the axis also becomes effective, indicating that repositioning can compensate for a lack of oscillation. Already, the very compact surface structure achieved with minimized depths in the valleys by the 2BA method supported the improvement in tribological performance and increase in load-carrying capacity, together with lubricant retention enhancement. These results show that the microfinishing process can be optimized by parameter tuning, and also, non-oscillating methods could come to be a practical alternative, probably reducing the complexity of equipment and cutting costs. Further studies need to be aimed at the scalability of these methods and their application to other materials and fields.

## 1. Introduction

**Figure 1:**
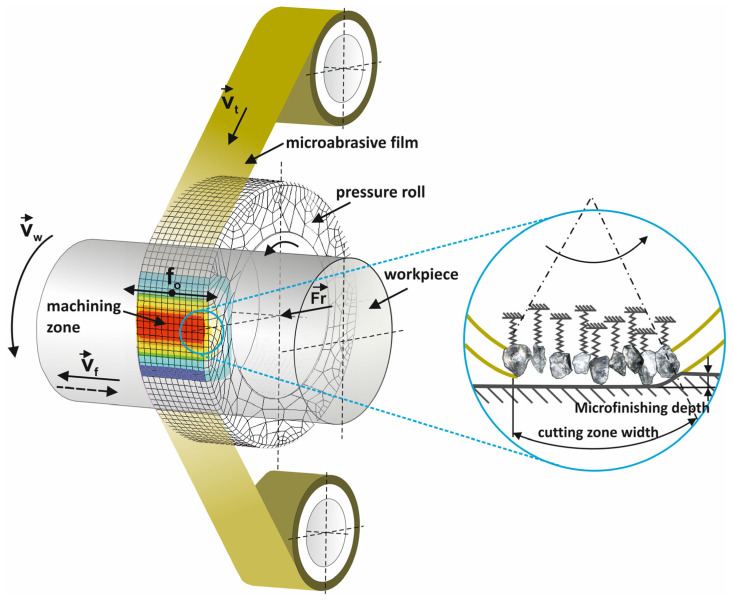
Kinematic diagram of rotary surface finishing using lapping films, where the following quantities are indicated on the diagram: *v_t_*—tool speed, *v_w_*—workpiece speed, *v_f_*—tool feed speed, *f_o_*—tool oscillation frequency, and *F_r_*—the pressure force of the pressing roller [15].

The microabrasive film superfinishing process was designed for cylindrical surfaces (Figure 1) but can be used for other shapes [1,2]. The demand for better surface finishes on parts is growing especially for precision applications and for the many materials that need to be finished [3,4]. Until the early 21st century the way to obtain precise surface finishes was with superfinishing stones. Now superfinishing with microabrasive film is the standard [5]. Compared to traditional finishing methods, superfinishing with microabrasive film reduces machining time and is a cost effective process and results in a precise and consistent finish across the entire surface regardless of part size [6,7]. Amorphous Ni–P alloy samples were used in this study because of their unique properties. Amorphous nickel–phosphorus (Ni–P) alloys have a unique combination of properties that make them suitable for many industrial applications [8,9]. These alloys are hard, wear resistant and corrosion resistant [10,11]. The absence of a crystalline structure in the amorphous Ni–P alloys gives them remarkable mechanical properties as it eliminates grain boundaries which are common sites for crack initiation and propagation [12,13,14]. The high phosphorus content in these alloys also makes them resistant to chemical attack so they can be used in harsh environments. Because of these properties amorphous Ni–P alloys are used in high performance and durability applications. Common applications are coatings for electronic components, automotive parts and precision instruments. In the electronics industry Ni–P coatings are used to protect printed circuit boards and connectors from corrosion and wear. In the automotive industry these coatings are used to extend the life of engine components and reduce friction. In precision engineering the wear resistance of Ni–P alloys ensures the longevity and reliability of the intricate mechanical parts.

**Figure 2:**
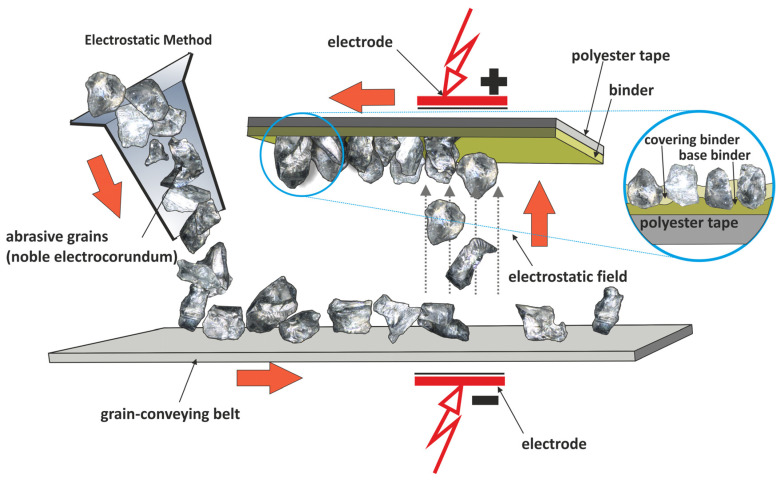
The production scheme in the electrostatic field of microfinishing films [37].

Basically, microfinishing films are coated abrasives [16,17]. The binder coating is smoothened at the top of the substrate made of polyester material, after which the holder abrasive grains are embedded into an electrostatic field as shown in Figure 2 [18,19]. In essence, this confirms that the abrasive grains are imbedded into the binder in a controlled manner, for that is the requirement for microfinishing [20,21]. Microfinishing films are used to obtain very fine surface finishes, which are needed in many high-precision engineering applications. The controlled deposition of abrasive grains ensures uniformity in the abrasive action and hence uniform surface texture and quality [22,23,24,25]. The abrasive grains become aligned perpendicular to the substrate due to the electrostatic field during its manufacturing (Figure 2). This approach maximizes each grain’s cutting efficiency, thereby reducing the time taken to achieve a desired surface finish [26,27,28]. The polyester substrate is also flexible and durable, thus allowing the microfinishing film to take any geometry of the surface without compromising its structure [29,30,31]. This is rather imperative, more so in the processing of complex shapes and contours, so that the entire surface is processed uniformly without missing any area [32,33]. Due to the efficiency that microfinishing films have over traditional abrasive tools, the facilities for many advantages in reducing material wastage and lowering operational cost, with better surface finish, are present [34,35,36]. These are very important in industries such as aerospace, automotive, and precision engineering, where the quality of the surface finish is related to the performance and life of the component directly. Microfinishing films represent one of the big developments in abrasive technology. Controlled, efficient, and uniform surface finishing makes them capable candidates for many manufacturing high-precision applications where enhanced performance and economy are sought.

Surface finish is very important for the performance and life of machined parts; therefore, microfinishing assumes a very important role in precision engineering [38,39]. Despite all the innovations carried out in conventional machining, producing parts with high surface smoothness is a challenging task, more so for high-precision and highly durable parts [38,40]. Microfinishing techniques using microabrasive films have been developed with the aim to enhance the surface finish, but the configuration of these new processes is yet to reach its full potential [41,42]. The main objective of this paper was to investigate the effect of different methods of microfinishing on surface topography to identify the most effective and efficient method so that high surface smoothness can be achieved. Specifically, the current study focused on researching the effects of different machining setups with and without oscillations on the surface finish of amorphous nickel–phosphorus Ni–P alloy workpieces. This material was chosen since it has no crystalline structure—ideally suited for observing the machining marks and thus could be more effective in assessing the performance of microabrasive films. At present, many developments are carried out in the area of microfinishing, although until now there has been a lack of understanding of the comparative effectiveness of the same process configuration [43,44]. This typical approach focuses on individual parameters, while comprehensive analysis, bringing together several roughness parameters and new assessment techniques like “analysis of island”, has not been conducted. Oscillation in the microfinishing process is required to obtain a uniform surface finish [45,46]; but it can be compensated for by repositioning the machining zone to simplify the whole process. The traditional method of machining along the workpiece axis with and without oscillation was compared, and a new approach of machining below the workpiece axis proposed in this paper. One of the primary objectives of the present work was to evaluate these configurations and find out an optimum set up for microfinishing processes. The results may bring important knowledge on how to optimize microfinishing techniques, decrease the complexity of a machine, and increase the quality of machined surfaces. It emerges from the results that proper setting of the machining parameters to achieve the best surface finish is among the most important factors relevant to the advancement of technology in precision engineering and manufacturing [47].

## 2. Materials and Methods

### 2.1. Microfinishing Process

**Figure 3:**
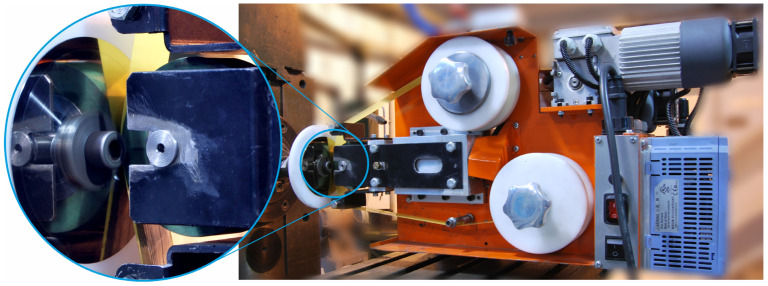
The research setup for the microfinishing process.

The microfinishing process utilizes a rather specialized microfinishing attachment designed to enhance the surface finish of the machined parts. A very special attachment, easily retrofitted to most conventional turning and milling machines, allowing for easy integration into existing manufacturing lines with special equipment, is all that is needed. Figure 3 was included to show the setup of a microfinishing attachment on a typical machine tool. The GW-1 microfinishing attachment was carefully designed to fit snugly in the tool post slot, ensuring maximum stability and precision throughout the operational process.

**Figure 4:**
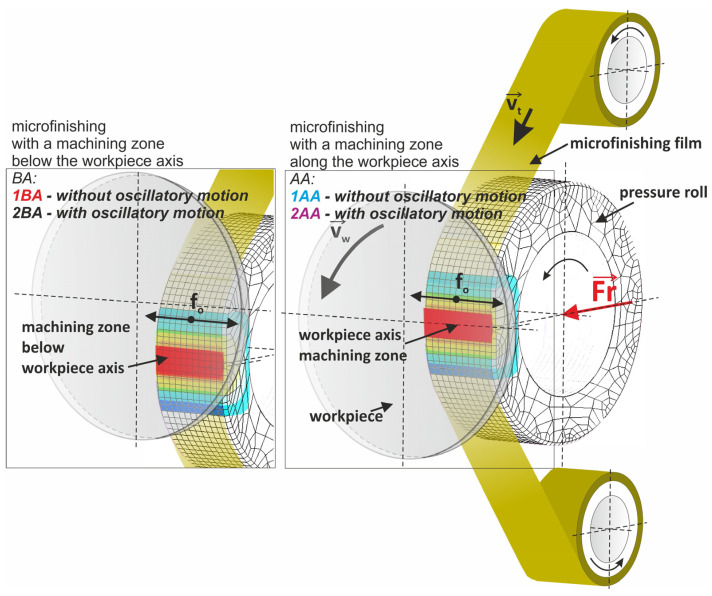
Four machining setups for the microsmoothing process: 1BA—microfinishing with a machining zone below the workpiece axis without oscillatory motion, 2BA—microfinishing with a machining zone below the workpiece axis with oscillatory motion, 1AA—microfinishing with a machining zone along the workpiece axis without oscillatory motion, 2AA—microfinishing with a machining zone along the workpiece axis with oscillatory motion.

Several key operating parameters control the microsmoothing process. These are feed rate (*vf*), oscillation frequency (*fo*), and roller pressure (*Fr*). The feed rate allows microabrasive films to be delivered at variable rates from 0 to 90 mm/min in order to adjust the material removal rate and surface finish to suit different materials and hardness. An oscillation frequency from 0 to 500 cycles per minute is key to obtaining a consistent and uniform finish by continually varying the contact points between the abrasive film and workpiece. A roller pressure from 10 to 90 N is applied to the workpiece via a pneumatic actuator to have a consistent and controllable force. The pneumatic system is powered by a 0.6 MPa pressure supply to enable precise control over the process. Physically the microfinishing attachment is compact at 575 mm long, 250 mm wide, and 300 mm high, so that it can be easily mounted and integrated into various machine tool setups without any modifications. It is also 25 kg weight so one person can handle and install it yet it is robust enough to withstand continuous industrial use. This microfinishing attachment is a versatile and efficient way to improve the surface finish of many machined components. It is compatible with standard machine tools and with precise control of the operating parameters and is an essential tool in precision engineering and manufacturing. The microfinishing was performed in the setup shown in Figure 4. The workpiece was a flat component. A press roller with 50 Sh hardness was applied to the workpiece with 50 N force. The abrasive film feed rate was 160 mm/min and the workpiece feed speed in the machining zone was 105 m/min—80 Hz oscillation frequency was applied for 20 s. This setup was designed to be optimal for microsmoothing considering roller hardness, pressure force, feed rates, oscillation frequency, and machining time. The flat workpiece geometry allowed for precise control and observation of the process in order to analyze the surface quality.

**Figure 5:**
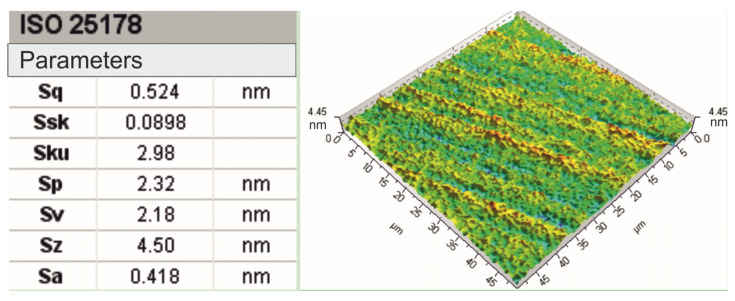
The super-smooth surface of the workpiece along with its surface roughness parameters for evaluation were determined according to the standard ISO 25178 [48].

In standard practice microfinishing is conducted along the axis of the workpiece. Microfinishing may be done with or without additional oscillation of the tool. Of course, the microfinishing attachment must be able to impart oscillation to the tool, which is often performed by an electric motor drive. Four microfinishing configurations were tested. The first two were standard, along the axis of the workpiece. The first one without additional oscillation of the tool is called 1AA in this article and the second one with oscillation of the tool is called 2AA. The third is a new approach to microfinishing where the machining zone is below the axis of the workpiece. This was also tested in two ways, one without oscillation of the tool, called 1BA, and the other with oscillation of the tool called 2BA, as shown in Figure 4. Microfinishing is a sequential machining process where the surface is polished with microabrasive films that contain increasingly finer grains to remove the machining marks left by previous operations. The risk of residual marks from previous treatments was reduced due to the fact that an initial surface was used, which was very smooth and mirror-like in quality, as shown in Figure 3, with a height of only up to 4.5 nanometers as visible in Figure 5. With a 30 μm grain size tool in the microfinishing process, this made the initial surface roughness close to zero. The workpiece processed was the amorphous nickel–phosphorus alloy (Ni–P). This was selected because this workpiece has no crystalline structure; hence, it is perfect for observing the machining marks throughout the micropolishing process. Apart from that, the material is hard, so it can be used to test the performance of microabrasive films. The surface topography before microfinishing was measured using the TalySurf CCI 6000 measurement system from Taylor Hobson, Leicester, UK.

**Figure 6:**
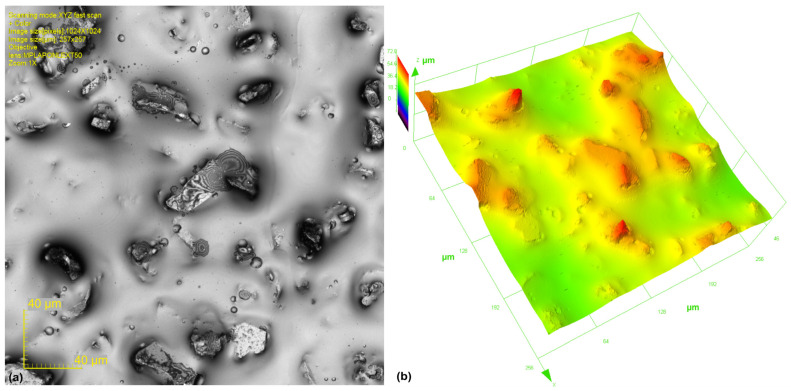
The surface of 30-micrometer nominal grain size microfinishing films, labeled as 30 MFF, was imaged using Olympus OLS4000 confocal microscope. 3D (**a**) and 2D (**b**) confocal images are 257 × 257 μm.

A microabrasive film with a nominal grain size of 30 μm was used for this microfinishing process (Figure 6). The abrasive grains used were of high purity electrocorundum. Since it is a microfinishing film, the abrasive grains will be embedded in a binder in an electrostatic field, and hence the abrasive particles are optimally orientated for the abrasive particles. Hence, this will give maximum material removal during the process. Topography of the microabrasive film surface was measured on an Olympus OLS4000 scanning confocal microscope from Tokyo, Japan.

### 2.2. Assessment of the Level of Surface Smoothness Being Processed

**Figure 7:**
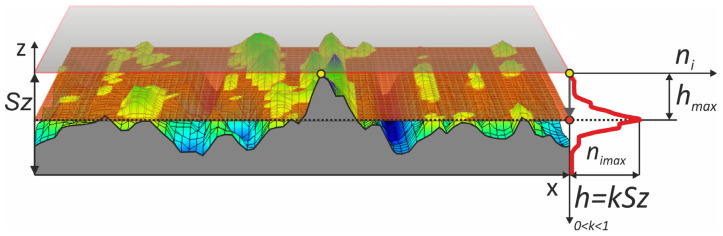
Methodology for surface evaluation using the “analysis of islands” technique.

The study used the TalySurf CCI6000 to measure four different microfinished surfaces. Each surface was measured multiple times using the stitching function on the machine to get full coverage. Each surface was measured over an area of 2.91 × 1 mm, 570 × 1658 points. For the analysis of machined surfaces in a 3D system, TalyMap Platinum 7.4 software (produced by Digital Surf, Besançon, France) was used. A new method to measure surface smoothness was introduced using the “analysis of islands” technique. For each surface an island analysis was carried out. A cut-off plane was set at the highest peak on the surface and then lowered by a value *h*, where *h* = *k*Sz, and Sz is the maximum height of the surface, until *Sz* is reached. The surface was measured at the following cut-off levels: *k* = 0.2, 0.25, 0.3, 0.35, 0.4, 0.45, 0.5, 0.55, 0.6, 0.8. At each cut-off level *n_i_* was calculated. For each surface *h_max_* was found where *n_i_* was maximum (Figure 7).

Next, the rate of change of the number of islands with respect to the position of the cut-off plane was calculated. This was done by dividing the change in the number of protrusions, *n_i_*, by the change in the cut-off plane, *h*. For each surface at the cut-off level *h_max_* where the most protrusions occur, we conducted a shape analysis of each protrusion. This shape analysis involved calculating the height/surface ratio, defined as the maximum height divided by the area. We also calculated the areas of the bases of all protrusions and plotted them. To quantify the level of surface smoothing we developed a coefficient, *c_e_* (1). Higher values of this coefficient mean better microfinishing. This coefficient is proportional to the maximum number of protrusions *n_i_* above the cut-off plane and the position of the cut-off plane where this maximum number of protrusions occurs. The position of the plane is *h_max_/Sz*:

(1) $$c_{e}=n_{i_{\max}}*\left(\frac{h_{\max}}{S_{z}}\right)$$

Additionally, a more conventional method was employed to assess the degree of surface smoothness. A series of parameters were determined to evaluate the roughness of the machined surface according to ISO 25178 standards, specifically focusing on height parameters [48]: Sp: maximum height of peaks; Sv: maximum height of valleys; Sz: maximum height of the surface; Sa: arithmetical mean height of the surface.

## 3. Results and Discussion

### 3.1. Analysis of Surface Smoothness Using the Analysis of Islands Technique

**Figure 8:**
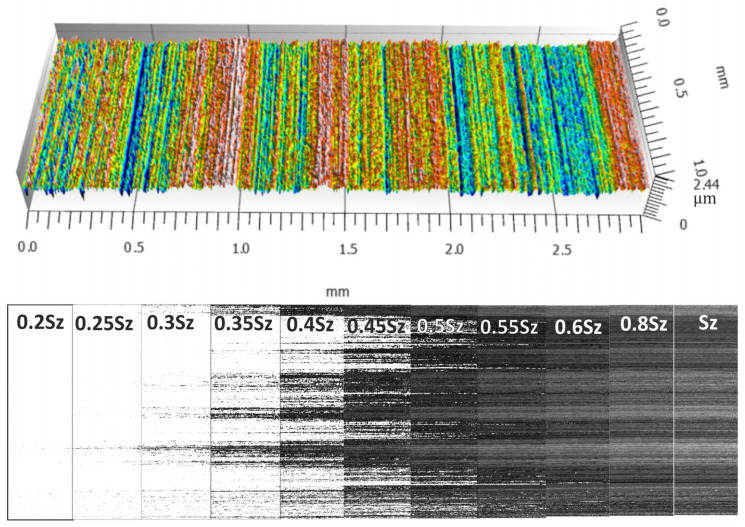
Surface topography in the machining setup 1AA, which is microfinishing with a machining zone along the workpiece axis without oscillatory motion, with protrusions above the cut-off plane at specific levels of *k*Sz marked in black.

**Figure 9:**
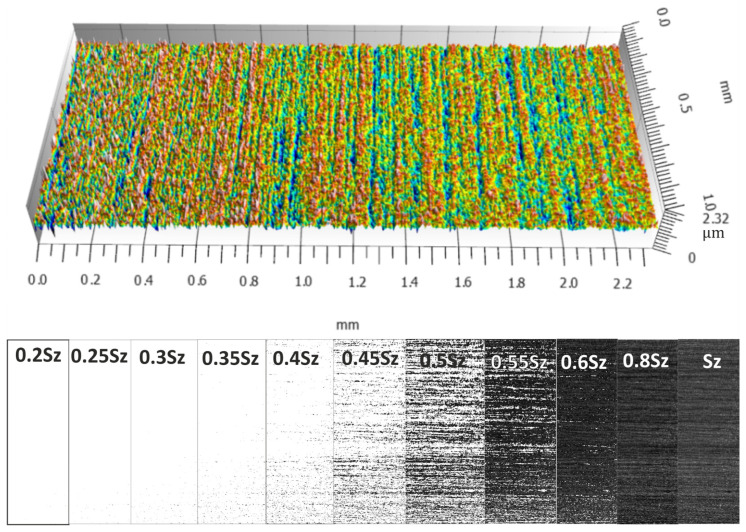
Surface topography in the machining setup 2AA, which is microfinishing with a machining zone along the workpiece axis with oscillatory motion, with protrusions above the cut-off plane at specific levels of *k*Sz marked in black.

**Figure 10:**
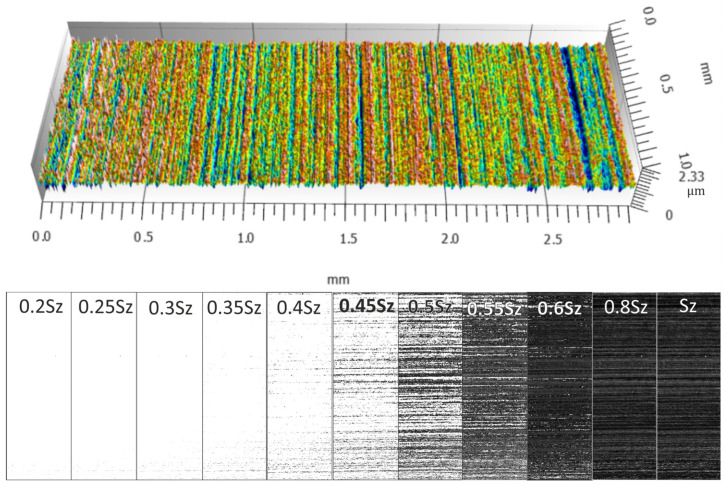
Surface topography in the machining setup 1BA, which is microfinishing with a machining zone below the workpiece axis without oscillatory motion, with protrusions above the cut-off plane at specific levels of *k*Sz marked in black.

**Figure 11:**
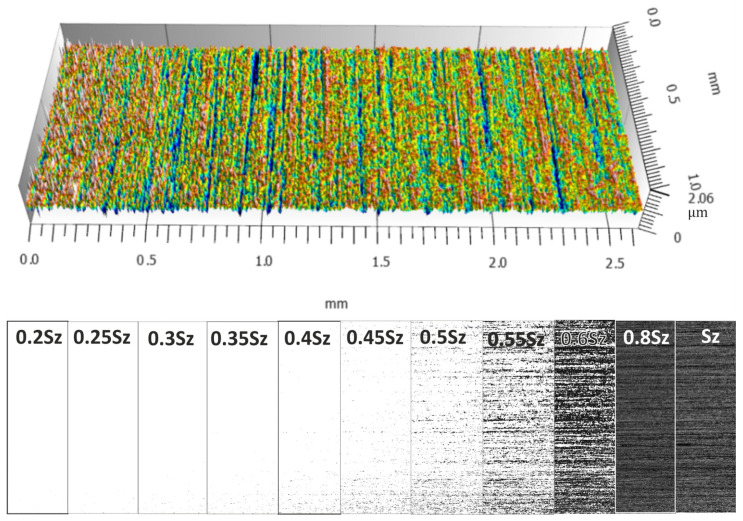
Surface topography in the machining setup 2BA, which is microfinishing with a machining zone below the workpiece axis with oscillatory motion, with protrusions above the cut-off plane at specific levels of *k*Sz marked in black.

Figure 8, Figure 9, Figure 10 and Figure 11 show the measured surfaces after four different microfinishing processes. The islands of material above the cut-off plane are shown at a distance of 0.2 Sz to Sz from the highest peak. The material above the cut-off plane is black in the figures. To obtain a complete picture we lowered the cut-off plane from the highest peak in steps of 0.05 Sz until Sz. This allows how the surface topography changes at different heights relative to the highest point to be seen. The black marked protrusions in the figures show the area where the surface is above the cut-off plane, in order to see how well the microfinishing process works in each variant.

By looking at the figures, you can see the distribution and density of the protrusions, which are indicators of surface roughness and smoothness. The comparison between the figures shows how each variant of the microfinishing process affects the surface texture and what surface quality is obtained with each method.

**Figure 12:**
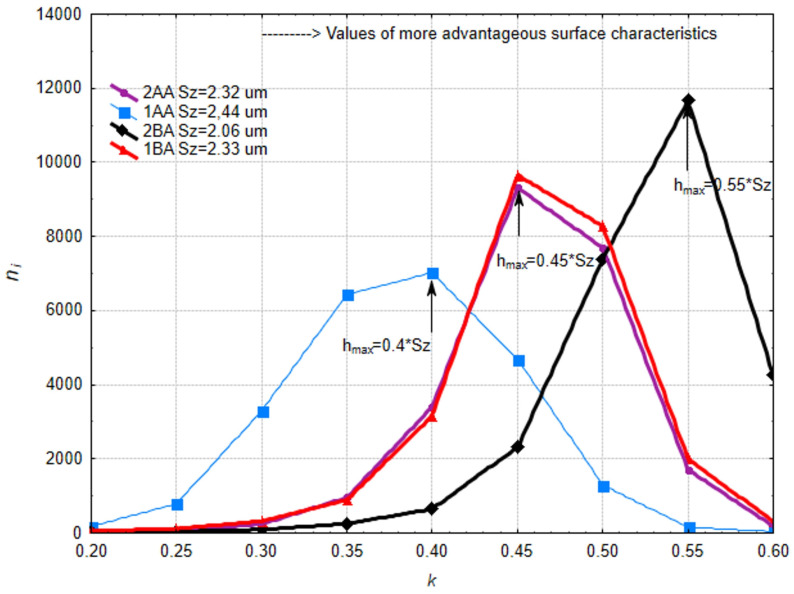
The number of protrusions *n_i_* above the cut-off plane at a distance *k* from the highest peak of the surface, which is the ratio of the distance *h* to the maximum surface height *Sz*.

For each surface after the microfinishing processes, the number of islands was counted at each cut-off level. Figure 12 shows the number of islands as a function of the cut-off plane position, expressed as the coefficient *k*, which is the ratio of the distance from the highest peak to the cut-off plane to the Sz. Sz is the maximum surface height. It can be seen that the machining setup with the processing zone along the workpiece axis without oscillatory motion has the fewest islands above the cut-off plane. Also, the maximum number of islands is reached the fastest, i.e., at the smallest distance from the highest peak of the surface. Since the initial surface was very smooth and microfinishing is designed to remove traces of previous machining processes, this is not good. For the two other surfaces, we have the same pattern. One was microfinished in the 1BA setup, where the machining zone is below the workpiece axis, and the other in the 2AA setup, where the machining zone is along the axis with oscillatory tool motion. In both cases we have the same maximum number of islands, *n_i_*, at the same hmax level, which is 0.45 Sz.

**Figure 13:**
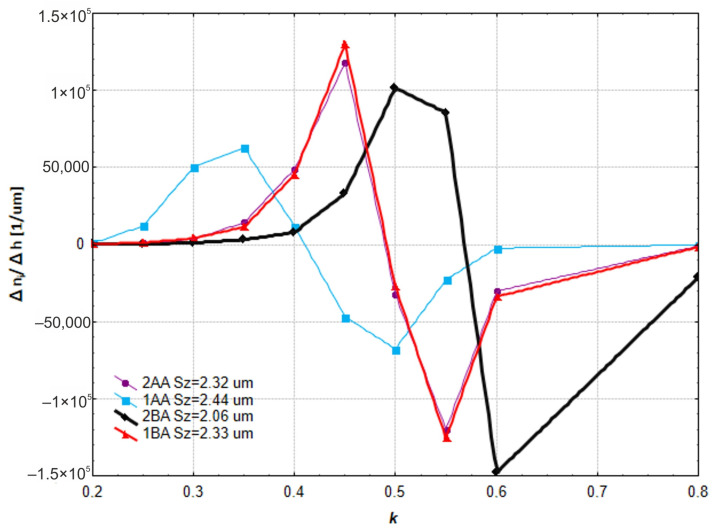
The ratio of the increase in the number of islands *n_i_* to the increase in the distance between consecutive levels *h* above the cut-off plane at a distance *k* from the highest peak of the surface, which is the ratio of the distance *h* to the maximum surface height Sz.

This is a great situation as it means we can reduce energy consumption and obtain the same surface finish using physical principles. Moving the microfinishing zone below the workpiece axis introduces an extra force in the machining zone which is not present in conventional machining. The best results were obtained for the 2BA setup where the microfinishing zone is below the workpiece axis and the tool is oscillating. This setup causes extra crosshatching of the tracks and the highest number of protrusions *n_i_*, which was over 10,000 at a cut-off plane distance of 0.55 Sz from the highest peak of the surface. This overall analysis shows that we need to optimize the microfinishing process by considering the position of the machining zone and the oscillating motion, both of which have a big impact on the surface finish. The results show we can obtain a big improvement on the surface finish by adjusting these parameters, and means we can get more efficient and better machining. The increase of islands vs. distance between consecutive cut-off plane levels was studied (Figure 13). The best topography of the protrusions is where the number of islands is maximum and has the highest increase of islands near the peak.

There is a big spread in the number of islands at different h levels which is not good. According to this criteria the best method is 2BA where the direction of the abrasive film and workpiece feed are not parallel, and oscillation is applied. This is followed by 2AA and 1BA which gave similar results. In 2AA the direction of the abrasive film and workpiece feed are parallel with oscillation and in 1BA the direction of the abrasive film and workpiece feed are not parallel but without oscillation. The worst method is 1AA, where the direction of the tool and workpiece feed are parallel without oscillation. A detailed analysis shows that the 2BA method is better because of the non-parallel motion of the tool and workpiece with oscillation which enhances the microfinishing by creating additional crosshatching of the surface. This crosshatching effect concentrates the islands at the optimal cut-off level and gives a better surface finish. Methods 2AA and 1BA are still good but do not give the same level of surface refinement because of the lack of either non-parallel motion or oscillation. Method 1AA is the worst because the foil and workpiece are parallel without oscillation which is not effective in microfinishing and the islands are not concentrated at any specific cut-off level so the surface topography is not good. The results show that the combination of non-parallel motion and oscillation in 2BA is the best.

**Figure 14:**
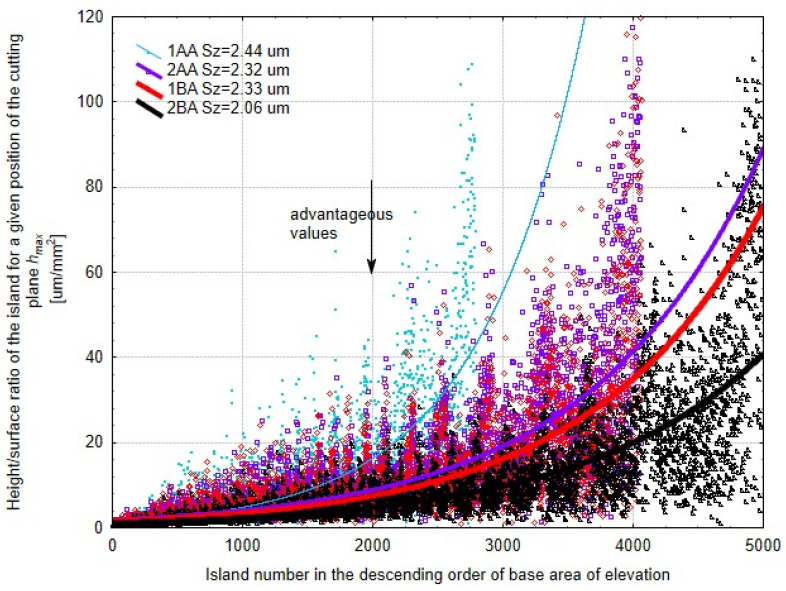
Height/surface ratio, defined as the ratio of the height to the base area of the protrusion for a specific cut-off plane position *h_max_*, where the number of protrusions is the highest. The results are arranged on the *x*-axis in descending order of contact area.

The islands above the cut-off plane were analyzed at *h_max_* position where the number of islands is highest. For each island the maximum height is above the cut-off plane and the base area of the island, i.e., the cross-section of the island formed by the cut-off surface (Figure 14).

It is better if the value is smaller, which means the protrusion above the cut-off plane is not narrow and tall. The smaller the value, the larger is the contact area of the machined surface in the practical applications of the produced surface. This method also proves the best surface structure is obtained by the 2BA method. Results of the 1BA and 2AA methods are similar, but 1BA is slightly better. So, we can skip the oscillation of the tool and shift the machining zone below the workpiece axis which is associated with simpler microfinishing attachments. A closer look reveals that a lower height/surface ratio means broader and shorter protrusions which is good for increasing the contact area in operational applications. This characteristic makes the surface more durable and functional. The 2BA method is better because it can produce a more stable and consistent surface structure due to the combination of non-parallel motion and oscillation. The 1BA method without oscillation is also good because by shifting the machining zone below the workpiece axis, it introduces an additional force during processing and produces a good surface structure without the need of a complex oscillatory mechanism. This means we can use a simpler microfinishing setup to produce a high quality surface as long as the machining zone is in the right position.

**Figure 15:**
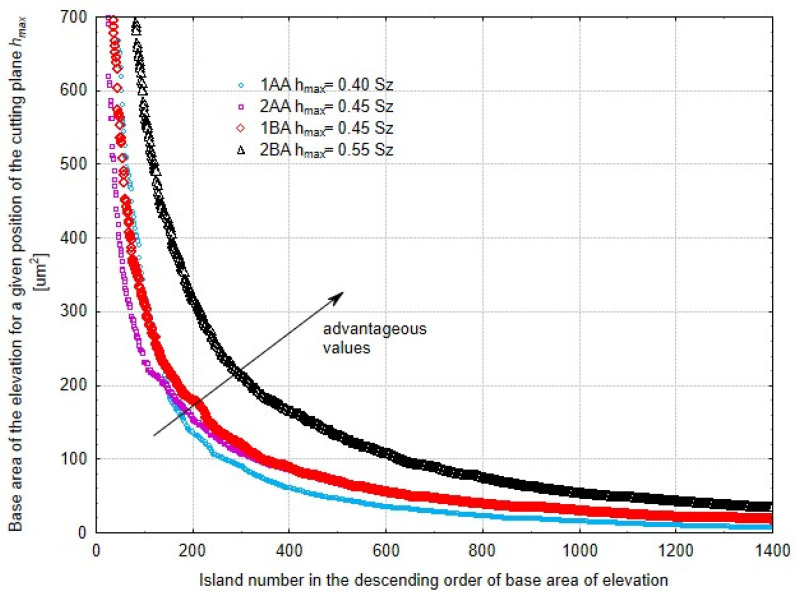
Base area of the protrusions for a specific cut-off plane position *h_max_*, where the number of protrusions is the highest. The results are arranged on the *x*-axis in descending order of contact area.

Figure 15 is the base area of the protrusion above the cut-off plane which is at a distance *h_max_* from the highest peak of the surface—this is the distance where the most protrusions occurred above this plane. A larger cross-sectional area of the protrusions is good because it will have significant tribological importance in the future. This is because of the increased sliding surface area between two interacting elements which makes the parts more durable and long-lasting. Again, the 2BA method is the best. High density of protrusions above the cut-off plane also means a good effect for the microabrasive film finishing process. The spaces between these protrusions can accumulate lubricant during the interaction of two elements which significantly improves the contact condition and extends the life of the components. This accumulation of lubricant in the inter-protrusion spaces reduces friction and wear and makes the mechanical performance more efficient and long-lasting. In a closer look, the 2BA method is better because of the combination of non-parallel motion and tool oscillation which produces a more consistent and robust surface structure. A larger base area of the protrusions created by this method gives more contact area which is important for load transfer and wear reduction. In addition, the surface topography produced by the 2BA method is better for lubricant retention which is critical in maintaining the surface integrity during operation. The method can produce a dense array of well-formed protrusions which means there is enough lubricant retention which minimizes friction and heat generation.

Based on the island data, an efficiency coefficient *c_e_* for microfinishing was calculated using Formula (1). Results are shown in Table 1. The higher the coefficient the better the surface is finished. The highest coefficient was for 2BA which agrees with our previous findings. This is the most efficient method; it has the highest surface density, which is good for the interaction with other elements and for the accumulation of lubricants in those spaces. Method 2BA is better because it creates a very compact surface structure which is good for load bearing and lubricant retention. This means better performance and longer life of the components in their operating environment. The efficiency coefficient is the key indicator of the microfinishing process and gives a quantifiable measure of the surface quality. Similar results were obtained for 1BA and 2AA which is very good. This means we can obtain the same machining results without tool oscillation, so the process is simpler. Method 1BA which does not use oscillation but still gets a dense and well-structured surface, which means repositioning the machining zone below the workpiece axis can replace the need for tool oscillation.

### 3.2. Analysis of Machined Surface Roughness

**Figure 16:**
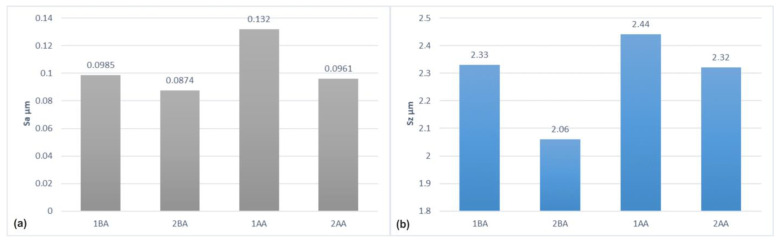
Parameters for assessing the surface roughness after four variants of surface smoothing: Sa—arithmetical mean height of the surface (**a**), Sz—maximum height of the surface (**b**).

The whole set of surface topographies after microfinishing was studied in order to obtain the surface roughness parameters for all methods. The most widely used parameter, Sa, the arithmetical mean height of the surface, supported the previous work with the technique of island analysis (Figure 16).

The lowest Sa value was for the 2BA method among these three methods, and 1BA and 2AA are nearly the same in the figure. Further, other roughness parameters, such as Sz, the maximum peak to valley height of the surface, supplemented the measure in which each microfinishing method was effective. The detailed analysis of Sa under the different machining processes revealed a better performance for the 2BA method in achieving a smoother surface finish. The matching between the Sa values and island analysis results verified the proper working of these techniques in the surface quality evaluation. The efficiency of the 2BA technique in giving the minimum Sa value highlighted well the capacity of this treatment to minimize surface defects, which is a prime requirement in industries that need a high level of accuracy and an exacting finish.

**Figure 17:**
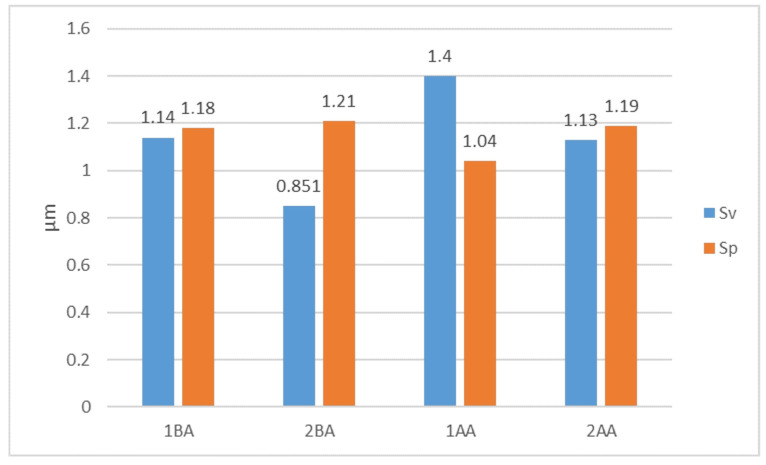
Parameters for assessing the surface roughness after four variants of surface smoothing: Sp—maximum height of peaks, Sv—maximum height of valleys.

The analysis of the surface roughness was extended about parameters Sv (maximum height of valleys), and Sp (maximum height of peaks) (Figure 17). Analysis of the parameter Sv is critical due to the deep scratches that form in the treatment on a smooth surface. As it was expected, the least effective method is the 1AA method, different from the other methods significantly; in this method, smoothing along the workpiece axis was introduced without oscillation. Here, the Sv value reaches up to 1.4 μm, while simultaneously having the lowest Sp value.

The lowest obtained Sv value was for the 2BA method, which has machining below the workpiece axis with oscillation, indicating an Sv value of 0.851 μm. The Sp, showing the height of peaks, was the highest in value but comparable to the 1BA and 2AA methods. Island analysis indicates that the peaks on the surface after the 2BA method are small and few in number and, therefore, will be rapidly removed either in subsequent machining operations or during the interaction of two elements through plastic deformation. Again, the 1BA and the 2AA methods give very similar results, further indicating that the oscillatory motion can be effectively eliminated while attaining similar microfinishing results. This analysis lends emphasis to the prime need for selecting appropriate machining parameters to optimize the microfinishing process for high–quality surfaces. Results for 1BA and 2AA methods, even without oscillatory motion, prove them to be effective in some applications. Hence, it may simplify the microfinishing process without much loss of quality.

## 4. Summary and Conclusions

The present study focused on effectiveness in terms of surface topography resulting from the different microfinishing techniques. It was found that the process involving machining below the axis of the workpiece with oscillatory motion resulted in the highest density of protrusions and lowest values of surface roughness. Roughness parameters also testified to minimal irregularities on the surface and optimal peak structure for the same method. In particular, the methods of no oscillation but the machining zone below the axis were found to show equally good effectiveness, meaning that repositioning compensates for the lack of oscillation, thus simplifying the process but at the same time retaining its benefits. It is this technique of “analysis of islands” that has put a high premium on surface structure achieved by these methods. Larger base areas of protrusions and higher densities, which improve tribological performance in terms of load-bearing capacity and improving lubricant retention, are the outcome. Hence, the study concludes that machining below the axis using oscillatory motion can be placed in the category of fruitful microfinishing techniques, significantly improving smoothness of the surface and structural integrity, with the non-oscillatory methods offering a viable alternative.

- The method of machining below the workpiece axis with oscillatory motion turned out to be the most effective from the viewpoint of improving the smoothness of a surface—in this case, it resulted in the largest density of protrusions and lowest values of roughness.
- Methods not using oscillatory motion but having the machining zone below the workpiece axis were equally good. This may imply that the relocation of the machining zone can substitute for lack of oscillation, hence making the process easier without sacrificing quality.
- The technique of “analysis of islands” showed that the method with machining below the axis by oscillatory motion ensures an excellent structure of a surface with a high concentration of islands at optimal cut-off levels.
- The best microfinishing technique indicated from the findings is the below-axis machining with oscillatory motion. However, the non-oscillatory techniques also offer some very promising alternatives, which bring flexibility to process optimization and quite likely reduce the complexity of equipment, hence the operational costs.

## Figures and Tables

**Table 1:** Surface smoothing efficiency coefficient.

Machining Variant	$n_{i_{max}}$	${\left(\frac{h}{Sz}\right)}_{n_{i_{max}}}$	c_e_
2AA	9310	0.45	4189.5
1AA	7023	0.4	2809.2
2BA	11,664	0.55	6415.2
1BA	9652	0.45	4343.4

## Data Availability

The original contributions presented in the study are included in the article, further inquiries can be directed to the corresponding authors.
